# Pathological laughter as prodromal manifestation of transient ischemic attacks—case report and brief review

**DOI:** 10.1186/s12883-015-0457-3

**Published:** 2015-10-12

**Authors:** Adriana O. Dulamea, Costel Matei, Ioana Mindruta, Virgil Ionescu

**Affiliations:** University of Medicine and Pharmacy “Carol Davila”, Bucharest, Romania; Neurology Department Fundeni Clinical Institute, Sos Fundeni nr 258, sector 2, Bucharest, 022328 Romania; Cardiology Department Emergency Institute for Cardiovascular Diseases “Prof. Dr. C. C. Iliescu”, Bucharest, Romania; Neurology Department University Emergency Hospital, Bucharest, Romania; Radiology Department Sanador Hospital, Bucharest, Romania

**Keywords:** Involuntary emotional expression disorder, Pathological laughter, Gelastic seizures, Transient ischemic attacks

## Abstract

**Background:**

Based on a case report, the authors reviewed the data about involuntary emotional expression disorder (IEED). IEED includes the syndromes of pathological laughing and crying (PLC) and emotional lability (EL). PLC is a rare disorder of emotional expression characterized by relatively uncontrollable episodes of laughter and crying or both that do not have an apparent motivating stimulus.

**Case presentation:**

Authors report the case of a 59-year-old man who presented with recurrent episodes of PLC of approximately 2 min duration, consisting of accelerated breathing, emission of guttural, snoring sounds, frowning of the eyebrows, followed by laughter accompanied by motor restlessness of all four limbs. PLC episodes preceded left carotid transient ischemic attacks (TIA’s) manifested by reversible aphasia and right hemiparesis. Electroencephalography performed during PLC episodes revealed no spike-wave activity. Brain magnetic resonance imaging showed lacunar infarcts in the left lenticulo-capsulo-thalamic area and multiple round lesions in the cortical-subcortical and in the deep white matter of frontal-parietal-occipital lobes bilaterally, with T2 hyperintensity, T1 isointensity and no diffusion changes. The episodes were interpreted as transient ischemic attacks although gelastic seizures could not be excluded. The etiological investigations revealed unstable plaques on the left carotid artery bulb and the aortic arch and a degenerative mitral valve stenosis. The patient was treated first with antiplatelet therapy and antiepileptic drugs but PLC stopped only after anticoagulation was started. During follow-up the patient continued to have left carotid and vertebrobasilar TIA’s being on oral anticoagulation. The patient became asymptomatic only after mitral valve replacement was performed.

**Conclusions:**

This case illustrates the difficulty distinguishing between gelastic epilepsy and TIA’s in cases of PLC episodes and discuss the neuroanatomic bases and pathophysiology of this rare condition.

**Electronic supplementary material:**

The online version of this article (doi:10.1186/s12883-015-0457-3) contains supplementary material, which is available to authorized users.

## Background

Involuntary emotional expression disorder (IEED) includes the syndromes of pathological laughing and crying (PLC) and emotional lability (EL) [1]. PLC is an exaggerated, uncontrollable and inappropriate laughter or crying usually unrelated to a true emotion or a congruent mood. An extremely rare form is the “fou rire prodromique” (prodrome of crazy laughter), a pathological manifestation reported in association to carotid or vertebrobasilar brain ischemia. Cases of PLC were described in association with cerebrovascular disease, amyotrophic lateral sclerosis, multiple system atrophy-cerebellar type, multiple sclerosis, Parkinson’s disease, traumatic brain injury, dementia, migraine, progressive supranuclear palsy and mass lesions especially in the cerebellopontine junction. PLC must be differentiated from emotional instability characteristic to pseudobulbar syndrome, from gelastic epilepsy and psychiatric disorders. We report the case of a man presenting recurrent pathological laughter episodes preceding transient ischemic attacks.

## Case presentation

A fifty-nine-year-old right-handed man, former smoker, with history of chronic alcohol intake, arterial hypertension, mixed hyperlipidemia, two recent left middle cerebral artery ischemic strokes (left temporo-insular and left frontal lacunar infarcts) manifested as right hemiparesis and aphasia, right focal motor seizures, gastric ulcer secondary to excessive use of nonsteroidal anti-inflammatory drugs for occipital headache was admitted for episodes of PLC started 6 h before admission. The episodes lasted approximately 2 min, consisting of accelerated breathing, emission of guttural, snoring sounds, frowning of the eyebrows, followed by laughter accompanied by motor restlessness of all four limbs; the patient was able to walk with assistance but was unresponsive during these episodes. An additional movie file shows this in more detail [see Additional file 1]. The patient denied any automatisms, loss of consciousness, loss of memory, or altered mental status. He also denies any sense of mirth during these episodes. On admission the patient’s home medication were clopidogrel, carbamazepin, gabapentin, pantoprazole, and quinapril. Initial examination was notable for confusion, right upper motor neuron facial weakness, 4/5 right hemiparesis, and a mixed aphasia. Between episodes of PLC there were pauses of about 10 min.

**Fig. 1:**
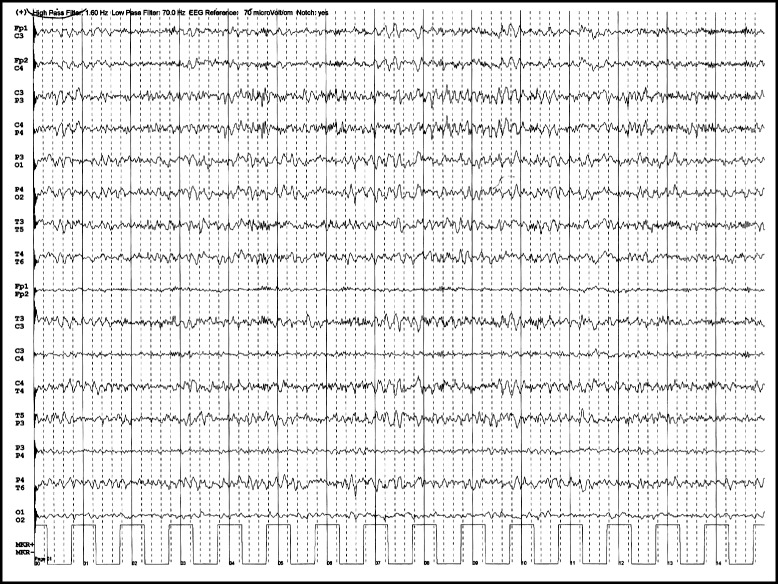
EEG performed during PLC episodes showed no spike wave activity only slow waves on the derivations on the right side

**Fig. 2:**
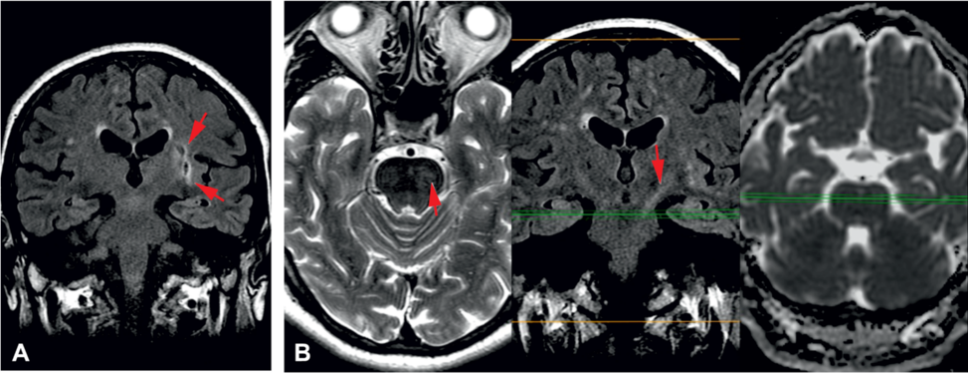
**A**, **B** Polygonal left side area intra- and supranuclear hyperintense T2, hypointense with hyperintense periphery on FLAIR images, which interface pyramidal fibers in the posterior arm of internal capsule leeding to Wallerian degeneration of them - left lateral pontine dots that apare hyperintense on T2 and FLAIR images, without restricted diffusion

**Fig. 3:**
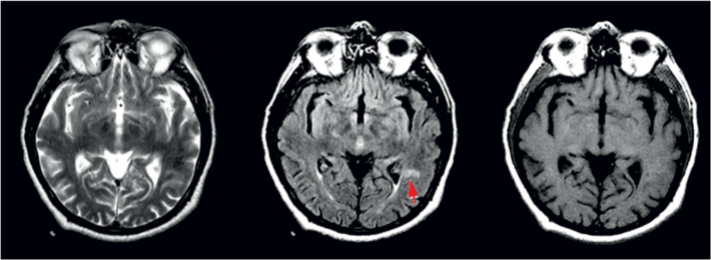
Paraventricular left temporal poligonal area with reduced dimensions between exams - area of ischemia in the subacute stage - hyperintense on T2 and FLAIR, hypointense on T1 without restricted diffusion

**Fig. 4:**
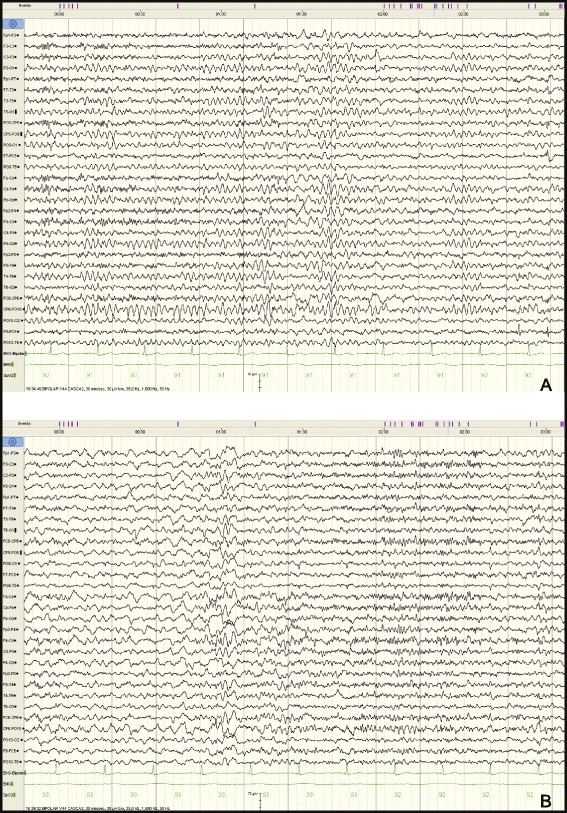
**A**, **B** Video EEG during awake state and during sleep without spike-waves anomalies but with some slow waves especially on the right side during sleep

The differential diagnosis was made between gelastic seizures and episodes of pathological laughter as manifestation of repeated transient ischemic attacks (TIA’s). The pseudobulbar laughter was excluded because the patient didn’t present any emotional incontinence outside the pathological laughter episodes. Brain scan showed left temporo-insular and left frontal chronic lacunar infarcts. Electroencephalography (EEG) performed during and after laughter episodes revealed no spike-wave activity (Fig. 1). Analysis of the cerebrospinal fluid specimen revealed a protein level of 0.3 g per liter, a glucose concentration of 72.8 mg per deciliter, a white blood cells count of 5 per cubic millimeter, polymorphonuclears of 2 per cubic millimeter, monocytes of 3 per cubic millimeter, no red blood cells. Routine laboratory blood test showed elevated uric acid of 8 mg per deciliter, slightly elevated gamma-glutamyl transferase of 57 U per liter, increased glucose level of 136 mmol per liter, increased lactate dehydrogenase of 236 mmol per liter, increased triglycerides of 152.3 mg per deciliter, increase white blood cells count of 10 790 per cubic millimeter with increased neutrophils blood count of 8 150 per cubic millimeter, all the other blood tests were normal. The patient was treated initially with clopidogrel, atorvastatin, diazepam and phenytoin during acute PLC episodes and clopidogrel, atorvastatin, carbamazepine 600 mg/day and levetiracetam 1000 mg/day as maintenance treatment, but the PLC episodes continued for 3 days with a shorter duration and a longer period between them. Brain magnetic resonance imaging (MRI), performed 5 days after symptoms onset, showed lacunar infarcts localized in the left lenticular-capsular-thalamic area associated with gliotic changes; multiple round lesions in the cortical-subcortical and in the deep white matter bilateral frontal-parietal-occipital, with T2 hyperintensity, T1 isointensity and no diffusion changes (Figs. 2 and 3). Based on symptoms, neurological examination and investigations the diagnosis of PLC as prodromal manifestation of TIA’s was made, heparin treatment was started and subsequently PLC episodes disappeared. However the gelastic seizures hypothesis could not be ruled out since EEG may not show spike-wave activity if the lesion is subcortical. Therefore the authors decided that antiepileptic treatment should be continued. The patient underwent a cognitive and psychological examination that did not reveal a cognitive dysfunction or a mood disorder. Carotid ultrasound showed non-hemodynamically significant plaque in the left carotid bulb. Transthoracic echocardiography showed moderate degenerative mitral stenosis (mitral valve area = 1.3 cm^2^, mean gradient 8 mmHg), mild mitral regurgitation, dilated left atrium, mild pulmonary hypertension and normal systolic left ventricular function and dimensions. Transesophageal echocardiography revealed, in addition to transthoracic echocardiography, left atrial spontaneous echo contrast (grade 3+), but no thrombus in left atrial appendage. Intra-cardiac defects with right-to-left shunt were also excluded. Some mobile atherosclerotic plaques on ascending aorta and initial part of the aortic arch were present, but no clear arguments for potential embolisation could be sustained. Given the degree of mitral stenosis, anticoagulation with acenocumarol was started, and clopidogrel was continued. Some concerns about this association were raised related to history of superior digestive hemorrhage of the patient, so clopidogrel 75 mg daily was preferred to low dose aspirin. The patient continued treatment with acenocumarol (INR = 2-3), clopidogrel 75 mg/day, atorvastatin 20 mg/day, carbamazepin 600 mg/day, gabapentin 900 mg/day and was discharged fully recovered. A video EEG was performed that showed no spike-waves only slow waves on the right side derivations (Fig. 4). A month later the patient started to present multiple left carotid and vertebrobasilar TIA’s despite correct anticoagulant treatment (INR in the therapeutic range). These ongoing embolic events led to treatment with mechanical valve replacement, afterwards the patient was completely asymptomatic.

## Discussion

Emotional experience is a subjective feeling during an emotional event. Emotional expression is the objective behavior that is expressed during such event, including changes in autonomic functions as heart rate and skeletal movements such as facial expression. The experience and the expression of an emotion depends on the cognitive appraisal of the emotional stimuli which are triggering it. While emotional experience is disturbed in patients with psychiatric conditions (e.g. mood disorders), patients with neurological disorders suffer from dysregulation of emotional expression, mostly the expressions of laughter or crying, in the absence of a congruent feeling. Unlike mood disorders, inappropriate emotional expression in these patients is not a sustained phenomenon but a paroxysmal and episodic one.

According to Poeck’s criteria [2], PLC is laughter that arises: (1) in response to non-specific stimuli; (2) in the absence of a corresponding change in affect; (3) in the absence of voluntary control of the extent or duration of the episode; and (4) in the absence of a corresponding change in mood lasting beyond the actual laughing. PLC is a disorder of emotional expression, not a disturbance of feelings, and can be distinguished from mood disorders [3].

To summarize the results of many studies investigating the neural correlates of laughter and humor, the expression of laughter seems to depend on two partially independent neuronal pathways [4]. The first of these, excitatory, “involuntary” or “emotionally driven” system, involves the amygdala, temporal cortex, thalamic/hypo- and subthalamic areas and the dorsal/tegmental brainstem. The second, inhibitory, “voluntary” system originates in the premotor/frontal opercular areas and leads through the motor cortex and pyramidal tract to the ventral brainstem [5].

These systems appear to be coordinated by a laughter-coordinating center in the dorsal upper pons, central coordinator of the nuclei that innervate the muscles involved in expression of emotion, phonation, rhythmic clonic expiration and facial expression. The mesencephalic central gray matter acts as relay station between descending limbic diencephalic tracts and bulbar effector nuclei and via the annulo-olivary tract to the cerebellum that exercises a modulating effect on all these expressions [6–8]. Parvizi et al. [6] suggested that cerebellum plays an important role in modulating laughter and crying to specific context; it also sets the threshold at which the induction – effector apparatus responds to a stimulus. These modulatory cerebellar actions would occur automatically as a result of learning.

The causes of PLC syndrome can be classified in two groups: altered behavior with unmotivated happiness (Angelman syndrome, schizophrenia, manias, dementia) and interference with the inhibitory/excitatory mechanisms (gelastic epilepsy, fou rire prodromique in strokes, multiple sclerosis, amyotrophic lateral sclerosis, Parkinson’s disease and Parkinson-plus, traumatic injuries, tumours) [9–11].

Gelastic epilepsy (from the Greek “gelos” meaning laughter) refers to those relatively rare seizures in which laughter is the cardinal symptom. These seizures can consist exclusively of laughing but often occur in association with general autonomic arousal and automatisms of movement and/or disturbed states of consciousness [12–17]. Several papers reported other symptoms accompanying less frequent this ictal laughter, such as perambulation [18] and micturition [19]. Some patients report pleasant feeling which include exhilaration or mirth [20–22], other patients experience the attacks of laughter as inappropriate and feel no positive emotions during their laughter [23–25]. The lesions incriminated in gelastic seizures are located in the hypothalamus especially hypothalamic hamartomas [14, 15, 24, 26–28], the frontal poles [21, 29] and the temporal poles [30]. Sethi and Rao [31] reported a patient with episodes of epileptic laughter, crying and running occurring alone or in combination. The patient was found to have a discrete, well circumscribed tumour of the left temporal lobe.

Fou rire prodromique [32] is a very rare condition in which unmotivated, inappropriate laughter occurs as the first symptom of cerebral ischemia. This uncontrollable laughter may be followed by giggling [33] or crying [34] and typical symptoms of stroke in the territory of carotid or basilar artery. Lesions associated with “fou rire prodromique” have been found in: (i) base of the pons bilaterally with no involvement of the tegmentum [33]; ii) left parahippocampal gyrus, the left posterolateral thalamus and adjacent parts of the internal capsule, with no involvement of the hypothalamus, the hippocampus or the amygdala [35]; (iii) left lenticular and caudate nuclei, with involvement of the anterior insula [36]; (iv) the area supplied by the right middle cerebral artery (non-dominant cerebral hemisphere) [37], (v) simultaneous bilateral capsular genu infarction [38], (vi) midbrain infarction [39], (vii) left middle cerebral artery territory (dominant cerebral hemisphere) [40], left pontine infarction due to basilar artery stenosis [41].

Parvizi J et al. (2007) [42] described a case of pathological laughter in the absence of congruent changes of mood in a patient with cerebellar type of multiple system atrophy (MSA-C). Nine patients out of 27 other patients with MSA-C revealed episode of pathological laughter, crying, or both. Authors finding of about 36 % occurrence suggests that the problem of dysregulation of emotional expression is more prevalent in MSA-C than the paucity of reports in the literature suggests and it is consistent with the view that the cerebellum and its interconnected structures may be involved in the regulation of emotional expression.

Olney N.T. et al. (2011) [43] compared amyotrophic lateral sclerosis (ALS) patients that presented episodes of PLC with ALS patients who did not have such episodes. The episodes were induced by contextually appropriate stimuli and associated with strong experiences of emotion that produced greater facial and physiological activation. The authors concluded that PLC represents activation of all channels of emotional responding (i.e. behavioural, physiological and subjective). Furthermore, they support previously advanced theories [44] that, rather than being associated with general emotional hyperreactivity, this disorder may be due to dysfunction in frontal neural systems that support voluntary regulation of emotion. Gallagher J.P. (1989) [45] examined 73 ALS patients of whom 36 had experienced episodes of PLC and nearly all of these developed bulbar involvement with the illness.

Cavanna A.E. et al. (2010) [46] described 8 patients with Gilles de la Tourette syndrome presenting PLC as part of their tic repertoire. All patients experienced PLC as a simple phonic tic, accompanied by characteristic premonitory urges and significant impairment in social interactions. The authors suggest that the pathophysiological mechanisms underlying the expression of PLC as a tic could involve a dissociation between fronto-striatal and limbic networks.

PLC is a component of global behavior pattern in Angelman syndrome. It has been suggested that the speech/language deficit in Angelman syndrome cannot be accounted for solely by mental retardation and that oral-motor dyspraxia and deficits in social interactions are quite characteristic of the syndrome. Peters S.U. et al. (2011) [47] showed that, in this syndrome, there is a decreased/delayed myelination, decreased axonal density diameter or aberrant axonal organization suggesting a generalized white matter alteration throughout the brain especially in temporal pathways and left arcuate fasciculus.

PLC was also described in Parkinson’s disease, multiple sclerosis, traumatic brain injuries, dementia, migraine, progressive supranuclear palsy [5], acute disseminated encephalomyelitis [48] or mass lesions (abscess in the anterior paramedian pons [49], cerebellar tumour [50], trigeminal schwannoma [51], frontal glioblastoma [52], pontine glioma [53]).

PLC in multiple sclerosis is the result either of isolated lesions to specific areas of the brain (in medulla oblongata and the mesencephalon [54], clinically isolated syndrome with pontine lesion [55]) or the consequence of an extensive diffuse involvement of the brain especially of the prefrontal cortex producing a complex neuropsychological impairment [56–58].

## Conclusions

This case illustrates the difficulty of distinguishing between TIA’s and gelastic seizures in cases of PLC. The absence of automatisms and spike-wave activity on EEG during episodes, the lack of efficacy of the antiepileptic treatment and the remission of episodes after anticoagulation and mitral valve replacement were arguments that supported the diagnosis of TIA’s. However a plausible assumption could be also that PLC episodes were generated by gelastic seizures caused by repeated ischemic episodes due to multiple TIA’s. In our patient the identification of multiple lacunar infarcts in the capsular area and cortical-subcortical frontal-parietal areas bilaterally as well as in the left pontine region is in concordance with the hypothesis of a complex system of emotion control which involves bilateral frontal and temporal lobes, the internal capsule, thalamic and subthalamic areas, upper brainstem, pons and cerebellum. PLC episodes have been reported in association with very diverse brain disorders. This case report showed that PLC is a rare clinical presentation of cardioembolic strokes.

### Patient consent

Written informed consent was obtained from the patient for publication of this case report and any accompanying images. A copy of the written consent is available for review by the editor of this journal.

## Additional files

Additional file 1:
**Episode of pathological laughter that preceded transient ischemic attacks in the territory of the left carotid artery.** (MP4 11.3 MB)

## Abbreviations

PLC: Pathological laughing and crying
IEED: Involuntary emotional expression disorder
EL: Emotional lability
TIA’s: Transient ischemic attacks
MRI: Magnetic resonance imaging
EEG: Electroencephalography
MSA-C: Cerebellar type of multiple system atrophy
ALS: Amyotrophic lateral sclerosis
